# Upstroke time is a more useful marker of atherosclerosis than percentage of mean arterial pressure for detecting coronary artery disease in subjects with a normal ankle-brachial index

**DOI:** 10.1038/s41440-024-01707-6

**Published:** 2024-05-17

**Authors:** Tatsuya Maruhashi, Masato Kajikawa, Shinji Kishimoto, Takayuki Yamaji, Takahiro Harada, Yu Hashimoto, Aya Mizobuchi, Shunsuke Tanigawa, Farina Mohamad Yusoff, Yukiko Nakano, Kazuaki Chayama, Ayumu Nakashima, Chikara Goto, Yukihito Higashi

**Affiliations:** 1 Department of Regenerative Medicine, Division of Radiation Medical Science, Research Institute for Radiation Biology and Medicine, Hiroshima University, 1-2-3 Kasumi, Minami-ku, Hiroshima, 734-8553 Japan; 2 Division of Regeneration and Medicine, Medical Center for Translational and Clinical Research, Hiroshima University Hospital, 1-2-3 Kasumi, Minami-ku, Hiroshima, 734-8551 Japan; 3 Department of Cardiovascular Medicine, Graduate School of Biomedical and Health Sciences, Hiroshima University, 1-2-3 Kasumi, Minami-ku, Hiroshima, 734-8551 Japan; 4 Department of Medicine and Molecular Science, Hiroshima University Graduate School of Biomedical Sciences, Hiroshima University, 1-2-3 Kasumi, Minami-ku, Hiroshima, 734-8551 Japan; 5 Department of Stem Cell Biology and Medicine, Graduate School of Biomedical and Sciences, Hiroshima University, 1-2-3 Kasumi, Minami-ku, Hiroshima, 734-8551 Japan; 6 Department of Rehabilitation, Faculty of general Rehabilitation, Hiroshima International University, 555-36, Kurosegakuendai, Higashihiroshima, 739-2695 Japan

**Keywords:** Ankle-brachial index, Cardiovascular risk, Percentage of mean arterial pressure, Upstroke time

## Abstract

Upstroke time (UT) and percentage of mean arterial pressure (%MAP) at the ankle have been shown to serve as atherosclerotic markers. The purpose of this study was to directly compare the diagnostic accuracy of UT with that of %MAP for clinical coronary artery disease (CAD) in subjects with a normal ankle-brachial index (ABI) in both legs. We measured UT and %MAP in 1953 subjects with a normal ABI. The optimal cutoff values of UT and %MAP derived from a receiver operating characteristic (ROC) curve to diagnose CAD were 148 ms and 40.4%, respectively. Multivariable analyses revealed that both UT ≥ 148 ms (odds ratio [OR], 2.72; *p* < 0.001) and %MAP ≥ 40.4% (OR, 1.28; *p* = 0.003) were significantly associated with CAD. When the subjects were divided into four groups according to the cutoff values of UT and %MAP, there was no significant difference in the risk of CAD between subjects with UT ≥ 148 ms and %MAP < 40.4% and those with UT ≥ 148 ms and %MAP ≥ 40.4% (OR, 1.45; *p* = 0.09). ROC curve analyses revealed that the area under the curve value of UT was significantly higher than that of %MAP (0.69 vs. 0.53, *p* < 0.001). The addition of UT to traditional risk factors significantly improved the diagnostic accuracy for CAD (0.82 to 0.84, *p* = 0.004), whereas the addition of %MAP to traditional risk factors did not improve the diagnostic accuracy for CAD (0.82 to 0.82, *p* = 0.84). UT is more useful than %MAP for identifying individuals with CAD among those with a normal ABI.

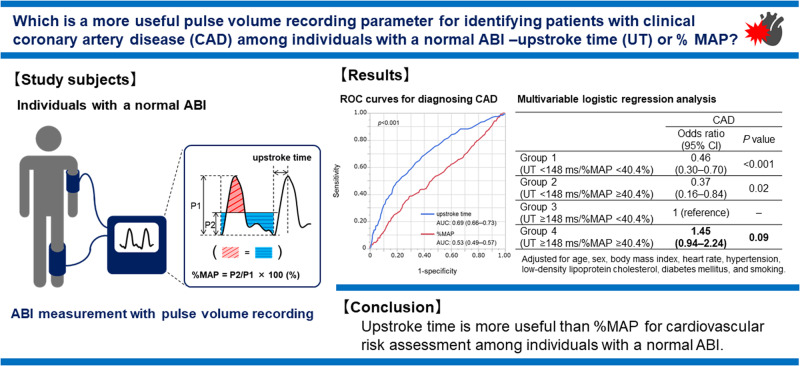

## Introduction

Ankle-brachial index (ABI) is the ratio of ankle systolic blood pressure to brachial systolic blood pressure [1]. ABI has been widely used not only for screening lower extremity arterial disease (LEAD) but also for cardiovascular risk assessment as a marker of generalized atherosclerosis and cardiovascular prognosis [2–6]. However, ABI can be unreliable in patients with severely calcified and non-compressible lower limb arteries since oscillometric ABI can be falsely elevated due to falsely elevated systolic blood pressure at the ankle, which can lead to the misclassification of patients with high cardiovascular risk as being at low cardiovascular risk [7–9]. Therefore, ABI should be used in combination with other vascular markers rather than ABI alone for cardiovascular risk assessment, particularly in individuals with a normal ABI.

Recent technological advancements in pneumoplethysmography using the new cuff method have enabled the automatic acquisition of high-quality pulse volume waveforms in a short time during ABI measurements. Upstroke time (UT) is the transit time from the nadir to peak of the pulse wave, and the percentage of mean arterial pressure (%MAP) is the height of the mean area of the arterial waveform divided by the peak amplitude [10]. These pulse volume recording parameters are automatically calculated and displayed immediately after ABI measurement, which can lead to the clinical utilization of UT and %MAP in the diagnosis of LEAD in combination with ABI. Indeed, the combination of ABI and pulse volume recording parameters has been shown to improve the diagnostic accuracy for LEAD compared with ABI alone [10]. In addition, recent studies have shown that pulse volume recording parameters may be useful not only in LEAD diagnosis but also for cardiovascular risk assessment as vascular markers of atherosclerosis [11–13]. The results of recent studies showed that both UT and %MAP were useful for identifying patients with clinical CAD [10, 13–15]. However, there is little information on the optimal cutoff values of UT and %MAP to diagnose clinical CAD and whether UT or %MAP is more useful for cardiovascular risk assessment in individuals with a normal ABI. Therefore, we investigated the optimal cutoff values of UT and %MAP to diagnose clinical CAD and directly compared the diagnostic accuracy of UT and that of %MAP for clinical CAD in a large number of well-characterized subjects with a normal ABI using an oscillometric device that can measure ABI, UT, and %MAP simultaneously.

Point of view

- **Clinical relevance:**
  The diagnostic accuracy of UT for CAD was superior to that of %MAP in individuals with a normal ABI, who are usually considered not to be at high cardiovascular risk by ABI measurement alone. Paying attention to UT may reduce the risk of missing patients with CAD in individuals with a normal ABI.
- **Future direction:**
  This study was a cross-sectional study. Further studies are needed to determine whether UT can serve as a more useful prognostic marker of cardiovascular events than %MAP in individuals with a normal ABI.
- **Consideration for the Asian population:**
  The device used for ABI measurement and pulse volume recording in this study has been widely adopted in Asia, particularly in East Asia, but not in Western countries.

## Methods

The data that support the findings of this study are available from the corresponding author on reasonable request.

### Subjects

This study was a cross-sectional study. Between January 2008 and December 2019, 2753 subjects were recruited for ABI measurement and pulse volume recording for cardiovascular risk assessment and LEAD screening from patients who visited the outpatient cardiology clinic and subjects who underwent health screening examinations with consent for vascular function assessment at Hiroshima University Hospital. Data were retrospectively analyzed. Some of the data have been previously reported elsewhere [13, 15, 16]. We excluded participants with severe aortic stenosis or aortic regurgitation (*n* = 35), atrial fibrillation (*n* = 181), LEAD defined as critical limb ischemia (*n* = 56), a history of major amputation (*n* = 55) or minor amputation (*n* = 12), or previous intervention including angioplasty or bypass graft (*n* = 76) and participants with missing information on a history of cardiovascular disease (*n* = 25). We further excluded participants with an ABI of either side <1.00 (*n* = 274), participants with an ABI of either side ≥1.40 (*n* = 85), and one participant with missing information on %MAP. Finally, 1953 participants (1197 men and 756 women; mean age: 60.8 ± 15.3 years) with bilateral normal ABI (1.0 ≤ ABI < 1.4) were enrolled in this study. Hypertension was defined as treatment with oral antihypertensive drugs or systolic blood pressure of more than 140 mm Hg and/or diastolic blood pressure of more than 90 mm Hg in a sitting position on at least 3 different occasions without medication [17]. Diabetes was defined according to the American Diabetes Association recommendation [18]. Dyslipidemia was defined according to the third report of the National Cholesterol Education Program [19]. We defined smokers as those who had ever smoked. CAD was defined as organic stenosis (≥50%) or occlusion of at least one coronary artery confirmed by coronary angiography (CAG), with or without a history of coronary revascularization procedures including percutaneous coronary intervention and/or coronary artery bypass grafting. The CAD diagnosis was confirmed by CAG in all patients with clinical CAD. However, not all subjects without clinical CAD underwent CAG. The exact number of subjects who were angiographically confirmed to have no CAD was unknown since information on whether the subjects underwent CAG was unavailable. Cerebrovascular disease included ischemic stroke, hemorrhagic stroke, and transient ischemic attack. ABI measurement and pulse volume recording were performed without withholding medications. This study was performed in accordance with the 1975 Declaration of Helsinki. The ethical committee of our institution (Hiroshima University Hospital Institutional Review Board) approved the study protocol. Written informed consent for participation in the study was obtained from all subjects.

### Study protocol

The subjects fasted the previous night for at least 8 h and abstained from consuming alcohol and caffeine and from smoking. The subjects were kept in the supine position in a quiet, dark, air-conditioned room (constant temperature of 22 °C–26 °C) throughout the study. A 23-gauge polyethylene catheter was inserted into the left deep antecubital vein to obtain blood samples. ABI measurement and pulse volume recording were performed at least 5 min after maintaining the supine position by skilled and trained physicians without detailed knowledge of baseline clinical characteristics of the subjects.

### ABI measurement and pulse volume recording

ABI measurement and pulse volume recording for UT and %MAP were performed by using a volume-plethysmographic apparatus as previously described (Form PWV/ABI, Omron Health Care Co., Kyoto, Japan) [13]. In brief, four oscillometric cuffs were wrapped around both upper arms and lower legs. Blood pressure in each limb was automatically and simultaneously measured. ABI was automatically calculated by dividing the ankle systolic blood pressure of the right and left sides by the higher brachial systolic blood pressure of either arm.

**Fig. 1:**
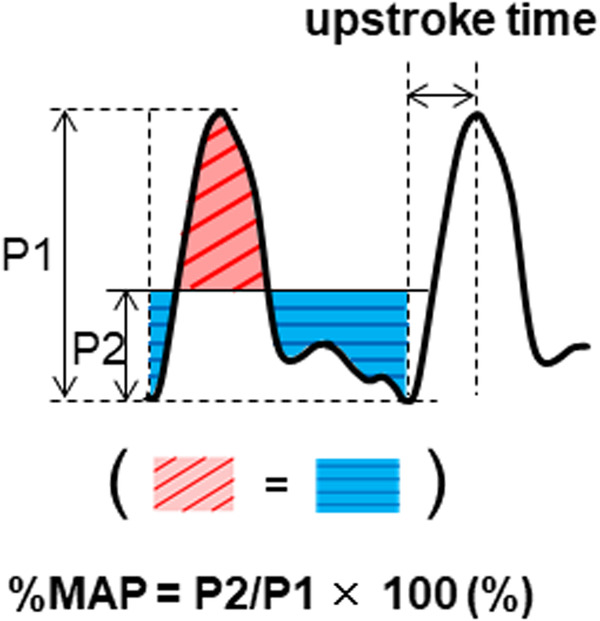
Upstroke time is the transit time from the nadir to the peak of the pulse wave. The percentage of mean arterial pressure (%MAP) is the height at which the enclosed pulse wave area is flattened (P2) divided by the peak amplitude (P1)

Waveforms of pulse volume recording were automatically obtained following the blood pressure measurement for ABI. Pulse volume waveforms in the lower limbs were recorded and stored for 10 s. The UT and %MAP were automatically calculated for each pulse waveform and the means of UTs and %MAPs obtained in the 10-second recording were used for analyses. UT is transit time from the nadir to peak of the pulse wave (Fig. 1). UT should be prolonged with hemodynamically significant stenosis or occlusion [10]. UT per cardiac cycle (UTCC) was calculated as the UT divided by cardiac cycle [UT × heart rate/600 (%)] [11, 12]. %MAP is the height at which the enclosed pulse wave area is flattened (P2) divided by the peak amplitude (P1) [P2/P1 × 100 (%)] (Fig. 1). The arterial waveform should be flattened and %MAP should increase with hemodynamically significant occlusive lesions in a lower extremity artery [10]. Additional details are available in the online Supplement.

### Assessment of intraobserver reproducibility of ABI, UT, and %MAP

Intraobserver reproducibility of ABI, UT, and %MAP was assessed in 26 men without clinical cardiovascular disease (mean age, 29.1 ± 9.9 years; age range, 20–50 years). ABI measurements and pulse volume recordings were performed twice at a four-week interval by the same experienced observer. We assessed the correlation between the first measurement and the second measurement and the coefficients of variation in ABI, UT, and %MAP.

### Statistical analysis

All reported probability values were 2-sided, and a *p* value of <0.05 was considered statistically significant. Continuous variables were summarized as means ± standard deviation (SD) and were compared by using unpaired or paired Student *t* test. Categorical variables were presented as frequencies and percentages and were compared by means of the chi-square test. To assess the diagnostic accuracy for clinical CAD, receiver operating characteristic (ROC) curve analyses were performed. Cutoff values of UT, UTCC, and %MAP were determined according to the highest Youden index from ROC curves to diagnose clinical CAD. The differences in area under the curve (AUC) were compared using the method of Delong et al. [20]. Multiple logistic regression analyses were performed to identify independent variables associated with CAD. Age, sex, body mass index (BMI), heart rate, hypertension, low-density lipoprotein cholesterol (LDL-C), diabetes mellitus, and smoking were entered as covariates into the model of the associations of CAD with UT and %MAP. Heart rate was not entered as a covariate into the model of the association of CAD with UTCC. Pearson’s correlation analysis was used to determine associations between vascular parameters in the first measurement and those in the second measurement. The data were processed using JMP version pro 16 (SAS Institute, Cary, NC).

## Results

### Baseline clinical characteristics

The baseline clinical characteristics of the subjects are summarized in Table 1. Of the 1953 subjects, 240 (12.3%) had CAD. The mean values for UT and %MAP were found to be 145.4 ± 22.8 ms and 38.5 ± 3.9%, respectively.

**Table 1:** Clinical characteristics of subjects according to upstroke time

Variables	All	Upstroke time <148 msec	Upstroke time ≥148 msec	*p* value
	(*n* = 1953)	(*n* = 1221)	(*n* = 732)	
Age, y	60.7 ± 15.2	58.6 ± 15.3	64.5 ± 14.5	<0.001
Male, *n* (%)	1197 (61.3)	807 (66.1)	390 (53.3)	<0.001
Body mass index, kg/m^2^	24.1 ± 3.9	24.0 ± 3.9	24.2 ± 4.0	0.16
Systolic blood pressure, mm Hg	132.4 ± 18.5	133.2 ± 18.3	130.9 ± 18.8	0.007
Diastolic blood pressure, mm Hg	78.7 ± 12.5	80.9 ± 12.1	75.1 ± 12.2	<0.001
Heart rate, bpm	68.9 ± 11.9	70.8 ± 12.0	65.6 ± 11.0	<0.001
Total cholesterol, mmol/L	4.97 ± 0.96	5.01 ± 0.95	4.90 ± 0.97	0.02
Triglycerides, mmol/L	1.60 ± 1.24	1.65 ± 1.30	1.52 ± 1.12	0.03
HDL cholesterol, mmol/L	1.54 ± 0.43	1.55 ± 0.43	1.52 ± 0.43	0.30
LDL cholesterol, mmol/L	2.87 ± 0.83	2.90 ± 0.83	2.83 ± 0.84	0.07
Glucose, mmol/L	6.23 ± 1.94	6.18 ± 1.85	6.33 ± 2.08	0.12
HbA1c, %	5.9 ± 0.9	5.8 ± 0.9	6.0 ± 0.8	0.005
Smoking, *n* (%)	1024 (52.7)	656 (54.0)	368 (50.6)	0.15
Comorbidities				
Hypertension, *n* (%)	1618 (82.8)	998 (81.7)	620 (84.7)	0.09
Dyslipidemia, *n* (%)	1393 (71.4)	830 (68.0)	563 (77.0)	<0.001
Diabetes mellitus, *n* (%)	508 (26.0)	256 (21.0)	252 (34.5)	<0.001
Coronary artery disease, *n* (%)	240 (12.3)	88 (7.2)	152 (20.8)	<0.001
Prior coronary intervention, *n* (%)	190 (9.7)	72 (5.9)	118 (16.1)	<0.001
Cerebrovascular disease, *n* (%)	130 (6.7)	67 (5.5)	63 (8.6)	0.007
Hemodialysis, *n* (%)	13 (0.7)	4 (0.3)	9 (1.2)	0.02
Medications, *n* (%)				
Antihypertensive drugs	1329 (68.0)	800 (65.5)	529 (72.3)	0.002
Lipid-lowering drugs	701 (35.9)	365 (29.9)	336 (45.9)	<0.001
Antidiabetic drugs	353 (18.1)	162 (13.3)	191 (26.1)	<0.001

bpm indicates beats per minute; *HDL* high-density lipoprotein, *LDL* low-density lipoprotein, *HbA1c* hemoglobin A1c

### Association between UT and CAD

The cutoff value of UT derived from the ROC curve to diagnose clinical CAD was 148 ms (AUC, 0.69; 95% confidence interval [CI], 0.66–0.73) (Supplementary Fig. 1A). The subjects were divided into two groups according to the cutoff value of UT: subjects with UT < 148 ms (*n* = 1221) and subjects with UT ≥ 148 ms (*n* = 732). The clinical characteristics of the subjects according to the cutoff value of UT are summarized in Table 1. The prevalence of CAD was significantly higher in subjects with UT ≥ 148 ms than in subjects with UT < 148 ms (20.8% vs. 7.2%, *p* < 0.001). Multiple logistic regression analysis revealed that UT ≥ 148 ms was significantly associated with clinical CAD after adjusting for confounding factors (odds ratio [OR], 2.72; 95% CI, 1.95–3.80; *p* < 0.001) (Supplementary Table 1). Every 1-SD increase in UT was significantly associated with an increased risk of clinical CAD (OR, 1.60; 95% CI, 1.38–1.86; *p* < 0.001) (Supplementary Table 1).

**Fig. 2:**
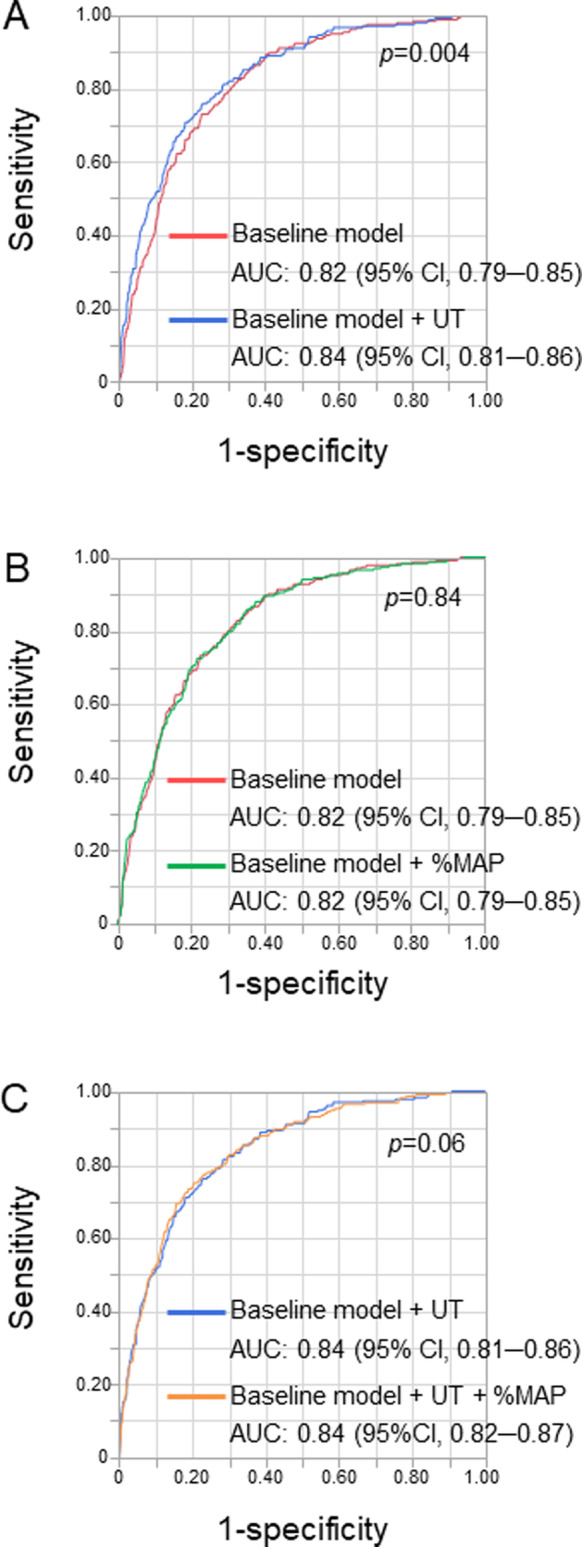
Receiver operating characteristic curves of the baseline model that comprises traditional cardiovascular risk factors and a combination of the baseline model and upstroke time (UT) to diagnose patients with coronary artery disease (CAD) (**A**), the baseline model and a combination of the baseline model and percentage of mean arterial pressure (%MAP) (**B**), and a combination of the baseline model and UT and a combination of the baseline model, UT, and %MAP (**C**). AUC indicates the area under the curve; CI confidence interval

The AUC value of the ROC curve for the baseline model that comprised traditional cardiovascular risk factors, including age, sex, BMI, hypertension, LDL-C, diabetes mellitus, and smoking, to diagnose clinical CAD was 0.82 (95% CI, 0.79–0.85) (Fig. 2A). The addition of UT to the baseline model significantly improved diagnostic accuracy for clinical CAD [AUC; 0.82 (95% CI, 0.79–0.85) to 0.84 (95% CI, 0.81–0.86), *p* = 0.004] (Fig. 2A).

### Association between %MAP and CAD

**Fig. 3:**
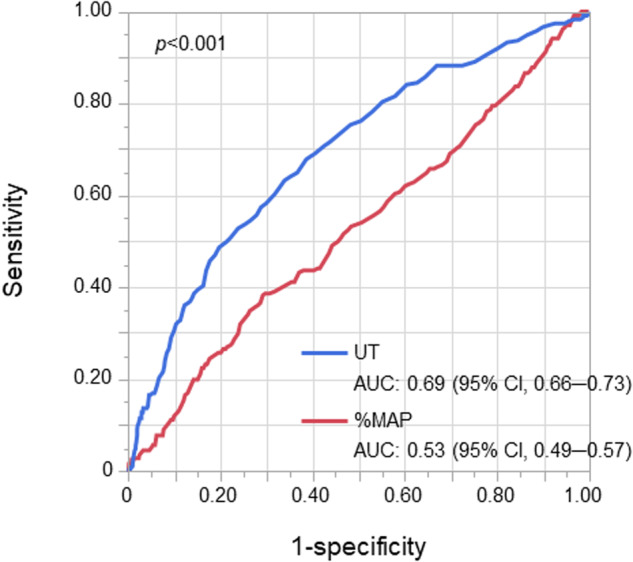
Receiver operating characteristic curves of upstroke time (UT) and percentage of mean arterial pressure (%MAP) to diagnose patients with coronary artery disease. AUC indicates the area under the curve; CI confidence interval

The cutoff value of %MAP derived from the ROC curve to diagnose clinical CAD was 40.4% (Supplementary Fig. 1B). The AUC value of %MAP to diagnose CAD was significantly lower than that of UT [AUC: 0.53 (95% CI, 0.49–0.57) vs. 0.69 (95% CI, 0.66–0.73), *p* < 0.001] (Fig. 3). The subjects were divided into two groups according to the cutoff value of %MAP: subjects with %MAP < 40.4% (*n* = 1362) and subjects with %MAP ≥ 40.4% (*n* = 591). The clinical characteristics of the subjects according to the cutoff value of %MAP are summarized in Table 2. The prevalence of CAD was significantly higher in subjects with %MAP ≥ 40.4% than in subjects with %MAP < 40.4% (15.6% vs. 10.9%, *p* = 0.004). Multiple logistic regression analysis revealed that %MAP ≥ 40.4% was significantly associated with clinical CAD after adjusting for confounding factors (OR, 1.85; 95% CI, 1.32–2.59; *p* < 0.001) (Supplementary Table 2). Every 1-SD increase in %MAP was significantly associated with an increased risk of clinical CAD (OR, 1.28; 95% CI, 1.09–1.50; *p* = 0.003) (Supplementary Table 2). The addition of %MAP to the baseline model comprising traditional cardiovascular risk factors did not improve diagnostic accuracy for clinical CAD [AUC; 0.82 (95% CI, 0.79–0.85) to 0.82 (95% CI, 0.79–0.85), *p* = 0.84] (Fig. 2B).

**Table 2:** Clinical characteristics of subjects according to percentage of mean arterial pressure

Variables	%MAP <40.4%	%MAP ≥40.4%	*p* value
	(*n* = 1362)	(*n* = 591)	
Age, y	60.3 ± 15.8	62.0 ± 15.7	0.02
Male, *n* (%)	910 (66.8)	287 (48.6)	<0.001
Body mass index, kg/m^2^	24.2 ± 3.9	23.7 ± 4.0	0.01
Systolic blood pressure, mm Hg	131.1 ± 17.9	135.2 ± 19.4	<0.001
Diastolic blood pressure, mm Hg	78.9 ± 12.3	78.3 ± 12.8	0.31
Heart rate, bpm	68.4 ± 12.2	69.9 ± 11.1	0.01
Total cholesterol, mmol/L	4.99 ± 0.97	4.91 ± 0.94	0.10
Triglycerides, mmol/L	1.64 ± 1.28	1.50 ± 1.12	0.03
HDL cholesterol, mmol/L	1.52 ± 0.41	1.59 ± 0.47	<0.001
LDL cholesterol, mmol/L	2.90 ± 0.83	2.80 ± 0.83	0.01
Glucose, mmol/L	6.15 ± 1.82	6.44 ± 2.18	0.004
HbA1c, %	5.8 ± 0.9	6.0 ± 0.8	0.02
Smoking, *n* (%)	732 (54.1)	292 (49.6)	0.07
Comorbidities			
Hypertension, *n* (%)	1135 (83.3)	483 (81.7)	0.39
Dyslipidemia, *n* (%)	976 (71.7)	417 (70.7)	0.66
Diabetes mellitus, *n* (%)	335 (24.6)	173 (29.3)	0.03
Coronary artery disease, *n* (%)	148 (10.9)	92 (15.6)	0.004
Prior coronary intervention, *n* (%)	121 (8.9)	69 (11.7)	0.06
Cerebrovascular disease, *n* (%)	91 (6.7)	39 (6.6)	0.95
Hemodialysis, *n* (%)	6 (0.4)	7 (1.2)	0.06
Medications, *n* (%)			
Antihypertensive drugs	935 (68.7)	394 (66.7)	0.39
Lipid-lowering drugs	468 (34.4)	233 (39.4)	0.03
Antidiabetic drugs	224 (16.5)	129 (21.8)	0.005

%MAP indicates the percentage of mean arterial pressure; *bpm* beats per minute, *HDL* high-density lipoprotein, *LDL* low-density lipoprotein, *HbA1c* hemoglobin A1c

%MAP (39.7 ± 3.6% vs. 37.8 ± 3.9%, p < 0.001) and the prevalence of %MAP ≥ 40.4% (40.2% vs. 24.0%, *p* < 0.001) were significantly higher in women than those in men. Therefore, we investigated the association between %MAP and CAD by sex to determine whether the difference in %MAP between men and women affects CAD detectability. In men, the prevalence of CAD was significantly higher in subjects with %MAP ≥ 40.4% than in subjects with %MAP < 40.4% (22.7% vs. 14.0%, *p* < 0.001). %MAP was significantly associated with CAD (OR, 1.81; 95% CI, 1.29─2.52; *p* < 0.001). In women, the prevalence of CAD was significantly higher in subjects with %MAP ≥ 40.4% than in subjects with %MAP < 40.4% (8.9% vs. 4.7%, *p* = 0.02). %MAP was significantly associated with CAD (OR, 2.00; 95% CI, 1.11─3.61; *p* = 0.02). These results suggest that, although the prevalence of %MAP ≥ 40.4% was higher in women than in men, the difference in %MAP between men and women did not affect CAD detectability.

### Associations between UT, %MAP, and CAD

The subjects were divided into four groups according to the cutoff values of UT and %MAP (Supplementary Fig. 2): subjects with UT < 148 ms and %MAP < 40.4% (Group 1, *n* = 1020), subjects with %MAP ≥ 40.4% and UT < 148 ms (Group 2, *n* = 201), subjects with UT ≥ 148 ms and %MAP < 40.4% (Group 3, *n* = 342), and subjects with UT ≥ 148 ms and %MAP ≥ 40.4% (Group 4, *n* = 390). The clinical characteristics of the subjects according to the cutoff values of UT and %MAP are summarized in Supplementary Table 3. There was a significant difference in the prevalence of clinical CAD among the 4 groups (7.8% vs. 4.0% vs. 19.9% vs. 21.5%, *p* < 0.001). When Group 3 was used as a reference, multiple logistic regression analysis revealed that Group 1 (OR, 0.46; 95% CI, 0.30–0.70; *p* < 0.001) and Group 2 (OR, 0.37; 95% CI, 0.16–0.84; *p* = 0.02) were significantly associated with a lower risk of clinical CAD, whereas there was no significant difference in the risk of CAD between Group 3 and Group 4 (OR, 1.45; 95% CI, 0.94–2.24; *p* = 0.09) (Table 3). The addition of %MAP to the combination of the baseline model and UT did not improve diagnostic accuracy for clinical CAD [AUC: 0.84 (95% CI, 0.81–0.86) to 0.84 (95% CI, 0.82–0.87), *p* = 0.06] (Fig. 2C).

**Table 3:** Associations of coronary artery disease with upstroke time and percentage of mean arterial pressure

Variable	Odds ratio (95% Confidence interval); *p* value		
	Unadjusted	Model 1^a^	Model 2^b^
Group 1 (UT < 148 ms/%MAP < 40.4%)	0.34 (0.24–0.49); <0.001	0.38 (0.26–0.55); <0.001	0.46 (0.30–0.70); <0.001
Group 2 (UT < 148 ms/%MAP ≥ 40.4%)	0.17 (0.08–0.36); <0.001	0.23 (0.11–0.51); <0.001	0.37 (0.16–0.84); 0.02
Group 3 (UT ≥ 148 ms/%MAP < 40.4%)	1 (reference)	1 (reference)	1 (reference)
Group 4 (UT ≥ 148 ms/%MAP ≥ 40.4%)	1.11 (0.77–1.58); 0.58	1.33 (0.90–1.97); 0.15	1.45 (0.94–2.24); 0.09

^a^Model 1: adjusted for age and sex

^b^Model 2: adjusted for age, sex, body mass index, heart rate, hypertension, low-density lipoprotein cholesterol, diabetes mellitus, and smoking

SD indicates standard deviation, *UT* upstroke time, %*MAP* percentage of mean arterial pressure

### Intraobserver reproducibility of ABI, UT, and %MAP

Intraobserver reproducibility was assessed by measuring ABI, UT, and %MAP twice with a four-week interval in 26 men. The clinical characteristics of the participants recruited for the assessment of intraobserver reproducibility are summarized in Supplementary Table 4. None of the subjects had hypertension, diabetes mellitus, clinical CAD, or clinical cerebrovascular disease, and none were treated with medications. Triglycerides were significantly decreased from 1.61 ± 1.47 mmol/L to 1.46 ± 0.72 mmol/L (*p* < 0.001). The other parameters including blood pressure and heart rate did not change. Pearson’s correlation coefficients of the first measurement and the second measurement were 0.68 for right ABI (*p* < 0.001), 0.71 for left ABI (*p* < 0.001), 0.50 for right UT (*p* = 0.01), 0.55 for left UT (*p* = 0.004), 0.16 for right %MAP (*p* = 0.44), and 0.37 for left %MAP (*p* = 0.07) (Supplementary Table 5). The coefficients of variation were 3.8 for right ABI, 3.3 for left ABI, 5.8 for right UT, 5.7 for left UT, 6.2 for right %MAP, and 5.4 for left %MAP (Supplementary Table 5). These results suggest that the reproducibility of %MAP was lower than that of UT.

### Association between UTCC and CAD

The cutoff value of UTCC derived from the ROC curve to diagnose clinical CAD was 17.0% (AUC, 0.57; 95% CI, 0.53–0.61). The subjects were divided into two groups according to the cutoff value of UTCC: subjects with UTCC < 17.0% (*n* = 1218) and subjects with UT ≥ 17.0% (*n* = 735). The clinical characteristics of the subjects according to the cutoff value of UTCC are summarized in Supplementary Table 6. The prevalence of CAD was significantly higher in subjects with UTCC ≥ 17.0% than in subjects with UTCC < 17.0% (15.7% vs. 10.3%, *p* < 0.001). Multiple logistic regression analysis revealed that UT ≥ 17.0% was significantly associated with clinical CAD after adjusting for confounding factors (OR, 1.46; 95% CI, 1.07–1.99; *p* = 0.02) (Supplementary Table 7). Every 1-SD increase in UTCC was not significantly associated with an increased risk of clinical CAD (OR, 1.14; 95% CI, 0.98–1.32; *p* = 0.08) (Supplementary Table 7). The AUC value of UTCC to diagnose CAD was significantly higher than that of %MAP [AUC: 0.57 (95% CI, 0.53–0.61) vs. 0.53 (95% CI, 0.49–0.57), *p* = 0.01]. However, the AUC value of UTCC to diagnose CAD was significantly lower than that of UT [AUC: 0.57 (95% CI, 0.53–0.61) vs.0.69 (95% CI, 0.66–0.73), *p* < 0.001]. These results suggest that UT is more predictive than UTCC of CAD.

## Discussion

In the present study, we investigated the optimal cutoff values of UT and %MAP from ROC curve analysis to diagnose clinical CAD and determined whether those cutoff values are useful for cardiovascular risk assessment in individuals with a normal ABI (1.0 ≤ ABI < 1.4). The results of the present study showed that the optimal cutoff values for UT and %MAP were 148 ms and 40.4%, respectively, and that these cutoff values were significantly associated with clinical CAD in individuals with a normal ABI. These findings suggest that UT and %MAP are useful for identifying patients with CAD in individuals with a normal ABI who are generally considered not to have advanced atherosclerosis by ABI measurement alone [3]. The optimal cutoff values of 148 ms for UT and 40.4% for %MAP to diagnose patients with CAD in individuals with a normal ABI are much lower than the recommended cutoff values of 180 ms for UT and 45.0% for %MAP for LEAD screening, indicating the possibility that UT values and %MAP values even below the recommended cutoff values for LEAD screening may be indicative of CAD in individuals with a normal ABI [10, 21, 22]. More attention should be paid to pulse volume recording parameters for more precise cardiovascular risk assessment in subjects with a normal ABI.

The ROC curve analyses revealed that the AUC value of UT for diagnosing CAD was significantly higher than that of %MAP and that the addition of UT to the baseline model that comprised traditional cardiovascular risk factors significantly improved the diagnostic accuracy for clinical CAD, whereas the addition of %MAP to the baseline model did not improve the diagnostic accuracy for clinical CAD. These findings suggest that UT is more useful than %MAP for cardiovascular risk assessment in individuals with a normal ABI. Furthermore, %MAP may have little incremental value in cardiovascular risk assessment when UT is considered. Multiple logistic regression analysis for the association between %MAP and clinical CAD showed that %MAP ≥ 40.4% was significantly associated with CAD, even after adjustment for confounders, indicating a significant association between %MAP ≥ 40.4% and CAD. However, when individuals were divided into four groups according to the cutoff values of UT and %MAP, only 201 of the 1953 individuals were divided into Group 2 (UT < 148 ms and %MAP ≥ 40.4%), and the prevalence of CAD was only 4.0% in Group 2, which was the lowest among the four groups. Multiple logistic regression analysis showed no significant difference in the risk of clinical CAD between subjects with UT ≥ 148 ms and %MAP < 40.4% and those with UT ≥ 148 ms and %MAP ≥ 40.4%. These findings suggest that the insignificance of %MAP ≥ 40.4% in cardiovascular risk assessment is more apparent when combined with UT < 148 ms. In addition, the ROC curve analyses showed that the addition of %MAP to the combination of the baseline model and UT did not improve the diagnostic accuracy for clinical CAD. These results suggest that %MAP has no incremental value in cardiovascular risk assessment when UT is considered in individuals with a normal ABI. To our knowledge, this is the first study to directly compare the diagnostic accuracy of UT and that of %MAP for clinical CAD as markers of atherosclerosis in individuals with a normal ABI and show that UT is more useful than %MAP for cardiovascular risk assessment.

However, the prevalence of clinical CAD was about 20% in patients with UT ≥ 148 ms among those with a normal ABI. Therefore, it may not be practical to conduct additional testing for CAD in all subjects with UT ≥ 148 ms and a normal ABI in terms of medical cost, medical staff manpower, and burden on patients. In subjects with UT ≥ 148 ms and a normal ABI, performing a careful physical examination, such as palpation of the lower limb arteries, and non-invasive tests, such as ultrasound imaging of the carotid artery, may be beneficial to select candidates for additional testing for CAD, especially in patients with traditional cardiovascular risk factors, including diabetes mellitus and chronic kidney disease, both of which are risk factors for calcified and non-compressible lower limb arteries.

Although we do not know the precise reasons for the superiority of UT to %MAP as a marker of atherosclerosis for cardiovascular risk assessment, the low reproducibility of %MAP may, in part, contribute to the inferiority of %MAP to UT for cardiovascular risk assessment. In the present study, a significant correlation was found between the first and second measurements in UT, but not in %MAP, indicating that the reproducibility of %MAP was lower than that of UT. Further studies are needed to clarify the reason for the superiority of UT to %MAP as a marker of atherosclerosis.

This study had some limitations. First, as this was a cross-sectional study, the possibility of residual unmeasured confounding factors cannot be excluded. Second, the results of the present study cannot be generalized to subjects with an ABI of either side <1.00 and subjects with an ABI of either side ≥1.40 since those subjects were excluded from the study. Third, not all subjects without clinical CAD underwent CAG. Therefore, we cannot deny the possibility that the subjects without clinical CAD had latent coronary artery stenosis. Fourth, it remains unclear whether the cutoff value of 148 ms for UT is useful for identifying patients at high risk for future cardiovascular events among individuals with a normal ABI. Further studies are needed to determine whether UT can be used as a prognostic vascular marker for future cardiovascular events. Fifth, interobserver variability was not assessed in this study. However, measurement of ABI and pulse volume recording using an oscillometric device are simple and less operator-dependent. Therefore, interobserver variability may not be high.

### Perspective of Asia

The device used in this study has been widely adopted in Asia, particularly in parts of East Asia. Measurements using this device are noninvasive, simple, less operator-dependent, and simple. Therefore, this device should be used more aggressively not only for LEAD screening but also for cardiovascular risk assessment in patients with cardiovascular risk factors.

## Conclusion

The diagnostic accuracy of UT for CAD was superior to that of %MAP in individuals with a normal ABI. UT may be more useful than %MAP as a marker of atherosclerosis for identifying patients with CAD among individuals with a normal ABI, who are usually considered not to be at high cardiovascular risk by ABI measurement alone. The optimal cutoff value of UT to diagnose clinical CAD is 148 ms, which is much lower than that of 180 ms recommended for LEAD screening. Paying attention to whether UT is greater than 148 ms may reduce the risk of missing patients with CAD in individuals with a normal ABI.

### Supplementary information

Supplementary information
