# Supplementary material for: Upstroke time is a more useful marker of atherosclerosis than percentage of mean arterial pressure for detecting coronary artery disease in subjects with a normal ankle-brachial index
Source: Hypertens Res. 2024 May 17;47(8):2009–18. doi: 10.1038/s41440-024-01707-6 (PMC11298401; doi:10.1038/s41440-024-01707-6)
Supplement: Supplementary file 1 — Supplementary information [file 41440_2024_1707_MOESM1_ESM.docx]

ONLINE SUPPLEMENT

**Upstroke time is a more useful marker of atherosclerosis than percentage of mean arterial pressure for detecting coronary artery disease in subjects with a normal ankle-brachial index**

Tatsuya Maruhashi, MD, PhD;^a^ Masato Kajikawa, MD, PhD;^b^ Shinji Kishimoto, MD, PhD;^a^ Takayuki Yamaji, MD;^c^ Takahiro Harada, MD;^a^ Yu Hashimoto, MD;^c^ Aya Mizobuchi, MS;^a^ Shunsuke Tanigawa, MS;^a^ Farina Mohamad Yusoff, MD;^a^ Yukiko Nakano, MD, PhD;^c^ Kazuaki Chayama, MD, PhD;^d^ Ayumu Nakashima, MD, PhD;^e^ Chikara Goto, PhD;^f^ Yukihito Higashi, MD, PhD^a, b^

^a^Department of Regenerative Medicine, Division of Radiation Medical Science, Research Institute for Radiation Biology and Medicine, Hiroshima University, 1-2-3 Kasumi, Minami-ku, Hiroshima, 734-8553, Japan

^b^Division of Regeneration and Medicine, Medical Center for Translational and Clinical Research, Hiroshima University Hospital, 1-2-3 Kasumi, Minami-ku, Hiroshima, 734-8551, Japan

^c^Department of Cardiovascular Medicine, Graduate School of Biomedical and Health Sciences, Hiroshima University, 1-2-3 Kasumi, Minami-ku, Hiroshima, 734-8551, Japan

^d^Department of Medicine and Molecular Science, Hiroshima University Graduate School of Biomedical Sciences, Hiroshima University, 1-2-3 Kasumi, Minami-ku, Hiroshima, 734-8551, Japan

^e^Department of Stem Cell Biology and Medicine, Graduate School of Biomedical and Sciences, Hiroshima University, 1-2-3 Kasumi, Minami-ku, Hiroshima, 734-8551, Japan

^f^Department of Rehabilitation, Faculty of general Rehabilitation, Hiroshima International University, 555-36, Kurosegakuendai, Higashihiroshima, 739-2695, Japan

**Correspondence**: Yukihito Higashi, MD, PhD, FAHA

Department of Regenerative Medicine,

Research Institute for Radiation Biology and Medicine, Hiroshima University

1-2-3 Kasumi, Minami-ku, Hiroshima, 734-8553, Japan

Phone: +81-82-257-5831 Fax: +81-82-257-5831

E-mail: [yhigashi@hiroshima-u.ac.jp](mailto:yhigashi@hiroshima-u.ac.jp)

**ABI measurement and pulse volume recording**

ABI measurement and pulse volume recording for calculation of upstroke time and %MAP were performed by using a volume-plethysmographic apparatus (Form PWV/ABI, Omron Health Care Co., Kyoto, Japan).^1^ Four oscillometric cuffs were wrapped around both upper arms and lower legs. The cuffs were connected to an oscillometric pressure sensor for measurements of blood pressure. Blood pressure in each limb was automatically and simultaneously measured. This device distinguished between pulses of the anterior tibial artery, posterior tibial artery, and peroneal artery by using frequency analysis and automatically selected and displayed the pulse with the highest oscillation. ABI was automatically calculated by dividing the ankle systolic blood pressure of the right and left sides by the higher brachial systolic blood pressure of either arm.

Waveforms of pulse volume recording were automatically obtained following the blood pressure measurement by holding cuff pressure at 54 mm Hg in subjects with diastolic blood pressure above 62 mm Hg and by holding cuff pressure at 8 mm Hg below diastolic blood pressure in subjects with diastolic blood pressure below 62 mm Hg to minimize the influence of cuff pressure on hemodynamics.^2^ Pulse waveforms in the lower limbs were recorded and stored for 10 seconds. The upstroke time and %MAP were automatically calculated for each pulse waveform and the means of upstroke times and %MAPs obtained in the 10-second recording were used for analyses. A beat with a pulse interval 25% shorter or longer than the previous beat interval was excluded due to the possibility of arrhythmia or body movement. The upstroke time and %MAP were not calculated when the number of available pulses for calculation was less than three.

Upstroke time is transit time from the nadir to peak of the pulse wave. Upstroke time should be prolonged with hemodynamically significant stenosis or occlusion.^1, 2^

The %MAP is the height at which the enclosed pulse wave area is flattened (P2) divided by the peak amplitude (P1) [P2/P1 × 100 (%)]. The arterial waveform should be flattened and %MAP should increase with hemodynamically significant occlusive lesions in the lower extremity artery.^2^

**References**

1. Maruhashi T, Kajikawa M, Kishimoto S, Hashimoto H, Takaeko Y, Yamaji T, Harada T, Hashimoto Y, Han Y, Aibara Y, Yusoff FM, Hidaka T, Chayama K, Nakashima A, Goto C, Kihara Y, Higashi Y. Upstroke time is a useful vascular marker for detecting patients with coronary artery disease among subjects with normal ankle-brachial index. *Journal of the American Heart Association*. 2020;9:e017139

2. Hashimoto T, Ichihashi S, Iwakoshi S, Kichikawa K. Combination of pulse volume recording (pvr) parameters and ankle-brachial index (abi) improves diagnostic accuracy for peripheral arterial disease compared with abi alone. *Hypertension research : official journal of the Japanese Society of Hypertension*. 2016;39:430-434

**Supplementary Table 1.** Association between coronary artery disease and upstroke time

|  | Odds ratio (95% Confidence interval);  *p* value | | |
| --- | --- | --- | --- |
| Variable | Unadjusted | Model 1^a^ | Model 2^b^ |
| Upstroke time <148 ms | 1 (reference) | 1 (reference) | 1 (reference) |
| Upstroke time ≥148 ms | 3.37 (2.55–4.47);  <0.001 | 3.27 (2.42–4.42);  <0.001 | 2.72 (1.95–3.80);  <0.001 |
| Every 1-SD (22.8 ms) increment of upstroke time | 1.81 (1.61–2.04);  <0.001 | 1.82 (1.59–2.08);  <0.001 | 1.60 (1.38–1.86);  <0.001 |

^a^Model 1: adjusted for age and sex.

^b^Model 2: adjusted for age, sex, body mass index, heart rate, hypertension, low-density lipoprotein cholesterol, diabetes mellitus, and smoking.

SD indicates standard deviation.

**Supplementary Table 2**. Association between coronary artery disease and percentage of mean arterial pressure

|  | Odds ratio (95% Confidence interval);  *p* value | | |
| --- | --- | --- | --- |
| Variable | Unadjusted | Model 1^a^ | Model 2^b^ |
| %MAP <40.4% | 1 (reference) | 1 (reference) | 1 (reference) |
| %MAP ≥40.4% | 1.51 (1.14–2.00);  0.004 | 1.76 (1.30–2.39);  <0.001 | 1.85 (1.32–2.59);  <0.001 |
| Every 1-SD (3.87%) increment of %MAP | 1.14 (0.99–1.31);  0.05 | 1.24 (1.07–1.43);  0.005 | 1.28 (1.09–1.50);  0.003 |

^a^Model 1: adjusted for age and sex.

^b^Model 2: adjusted for age, sex, body mass index, heart rate, hypertension, low-density lipoprotein cholesterol, diabetes mellitus, and smoking.

%MAP indicates percentage of mean arterial pressure; SD, standard deviation.

**Supplementary Table 3**. Clinical characteristics of subjects according to the cutoff values of upstroke time and percentage of mean arterial pressure

|  |  | Group 1  (n = 1020) | Group 2  (n = 201) | Group 3  (n = 342) | Group 4  (n = 390) |  |
| --- | --- | --- | --- | --- | --- | --- |
|  | Upstroke time | <148 ms | <148 ms | ≥148 ms | ≥148 ms |  |
| Variables | %MAP | <40.4% | ≥40.4% | <40.4% | ≥40.4% | *p* value |
| Age, y | | 58.7 ± 15.1 | 57.6 ± 16.4 | 64.8 ± 14.2 | 64.2 ±14.9 | <0.001 |
| Male, n (%) | | 711 (69.7) | 96 (47.8) | 199 (58.2) | 191 (49.0) | <0.001 |
| Body mass index, kg/m^2^ | | 24.0 ± 3.8 | 23.6 ± 4.2 | 24.8 ± 4.0 | 23.8 ± 3.9 | 0.001 |
| Systolic blood pressure, mm Hg | | 132.4 ± 18.0 | 137.2 ± 18.9 | 127.3 ± 17.1 | 134.1 ± 19.6 | <0.001 |
| Diastolic blood pressure, mm Hg | | 80.8 ± 12.2 | 81.5 ± 11.7 | 73.4 ± 10.9 | 76.6 ± 13.0 | <0.001 |
| Heart rate, bpm | | 70.2 ± 12.2 | 73.8 ± 10.3 | 63.0 ± 10.4 | 67.9 ± 10.9 | <0.001 |
| Total cholesterol, mmol/L | | 5.02 ± 0.96 | 4.95 ± 0.89 | 4.92 ± 0.98 | 4.88 ± 0.97 | 0.11 |
| Triglycerides, mmol/L | | 1.67 ± 1.34 | 1.54 ± 1.10 | 1.56 ± 1.10 | 1.48 ± 1.14 | 0.07 |
| HDL cholesterol, mmol/L | | 1.53 ± 0.42 | 1.63 ± 0.49 | 1.48 ± 0.40 | 1.57 ± 0.46 | <0.001 |
| LDL cholesterol, mmol/L | | 2.92 ± 0.84 | 2.80 ± 0.77 | 2.86 ± 0.81 | 2.80 ± 0.86 | 0.06 |
| Glucose, mmol/L | | 6.16 ± 1.83 | 6.29 ± 1.92 | 6.11 ± 1.78 | 6.53 ± 2.31 | 0.02 |
| HbA1c, % | | 5.8 ± 0.9 | 5.7 ± 0.5 | 5.9 ± 0.7 | 6.1 ± 0.9 | <0.001 |
| Smoking, n (%) | | 564 (55.6) | 92 (45.8) | 168 (49.6) | 200 (51.6) | 0.03 |
| Comorbidities | |  |  |  |  |  |
| Hypertension, n (%) | | 835 (82.9) | 163 (81.1) | 300 (87.7) | 320 (82.1) | 0.07 |
| Dyslipidemia, n (%) | | 705 (69.1) | 125 (62.2) | 271 (73.2) | 292 (75.1) | <0.001 |
| Diabetes mellitus, n (%) | | 215 (21.1) | 41 (20.4) | 120 (35.1) | 132 (33.9) | <0.001 |
| Coronary artery disease, n (%) | | 80 (7.8) | 8 (4.0) | 69 (19.9) | 84 (21.5) | <0.001 |
| Prior coronary intervention, n (%) | | 66 (6.5) | 6 (3.0) | 55 (16.1) | 63 (16.2) | <0.001 |
| Cerebrovascular disease, n (%) | | 56 (5.5) | 11 (5.5) | 35 (10.3) | 28 (7.2) | 0.02 |
| Hemodialysis, n (%) | | 3 (0.3) | 1 (0.5) | 3 (0.9) | 6 (1.5) | 0.07 |
| Medications, n (%) | |  |  |  |  |  |
| Antihypertensive drugs | | 676 (66.3) | 124 (61.7) | 259 (75.7) | 270 (69.2) | <0.001 |
| Lipid-lowering drugs | | 310 (30.3) | 55 (27.4) | 158 (46.2) | 178 (45.6) | <0.001 |
| Antidiabetic drugs | | 136 (13.3) | 26 (12.9) | 88 (25.7) | 103 (26.4) | <0.001 |

%MAP indicates percentage of mean arterial pressure; bpm, beats per minute; HDL, high-density lipoprotein; LDL, low-density lipoprotein; HbA1c, hemoglobin A1c.

**Supplementary Table 4.** Clinical characteristics of subjects recruited for the assessment of intraobserver reproducibility

| Variables | First measurement | Second measurement (after 4 weeks) | *p* value |
| --- | --- | --- | --- |
| Age, y | 29.1 ± 9.9 | – | – |
| Men, n (%) | 26 (100) | – | – |
| Body mass index, kg/m^2^ | 21.8 ± 2.0 | 21.9 ± 1.9 | 0.23 |
| Systolic blood pressure, mm Hg | 119.3 ± 9.8 | 119.5 ± 8.6 | 0.92 |
| Diastolic blood pressure, mm Hg | 69.0 ± 9.6 | 69.7 ± 9.2 | 0.46 |
| Heart rate, bpm | 63.0 ± 10.3 | 64.8 ± 13.0 | 0.33 |
| Total cholesterol, mmol/L | 4.49 ± 0.89 | 4.68 ± 0.95 | 0.13 |
| Triglycerides, mmol/L | 1.61 ± 1.47 | 1.46 ± 0.72 | <0.001 |
| HDL cholesterol, mmol/L | 1.45 ± 0.36 | 1.47 ± 0.41 | 0.72 |
| LDL cholesterol, mmol/L | 2.49 ± 0.83 | 2.66 ± 0.82 | 0.15 |
| Glucose, mmol/L | 5.09 ± 0.46 | 5.27 ± 0.49 | 0.10 |
| HbA1c, % | 5.4 ± 0.3 | 5.4 ± 0.3 | 0.80 |
| Comorbidities |  |  |  |
| Hypertension, n (%) | 0 (0) | – | – |
| Dyslipidemia, n (%) | 11 (42.3) | – | – |
| Diabetes mellitus, n (%) | 0 (0) | – | – |
| Coronary artery disease, n (%) | 0 (0) | – | – |
| Cerebrovascular disease, n (%) | 0 (0) | – | – |
| Medications, n (%) | 0 (0) | – | – |

bpm indicates beats per minute; HDL, high-density lipoprotein; LDL, low-density lipoprotein; HbA1c, hemoglobin A1c.

**Supplementary Table 5.** Intraobserver reproducibility of ABI, upstroke time, and %MAP

| Variables | Before | 4 weeks | Pearson’s correlation  coefficient (r) | *p* value | CV |
| --- | --- | --- | --- | --- | --- |
| Right ABI | 1.15 ± 0.07 | 1.11 ± 0.08 | 0.68 | <0.001 | 3.8 |
| Left ABI | 1.15 ± 0.07 | 1.12 ± 0.08 | 0.71 | <0.001 | 3.3 |
| Right upstroke time (ms) | 135.0 ± 10.7 | 140.1 ± 9.2 | 0.50 | 0.01 | 5.8 |
| Left upstroke time (ms) | 135.7 ± 15.2 | 135.8 ±2 3.6 | 0.55 | 0.004 | 5.7 |
| Right %MAP (%) | 35.6 ± 3.1 | 36.2 ± 3.3 | 0.16 | 0.44 | 6.2 |
| Left %MAP (%) | 37.2 ± 3.0 | 36.8 ± 3.4 | 0.37 | 0.07 | 5.4 |

%MAP indicates percentage of mean arterial presure; ABI, ankle-brachial pressure; CV, coefficients of variation.

**Supplementary Table 6**. Clinical characteristics of subjects according to the cutoff values of upstroke time per cardiac cycle

|  | UTCC  <17.0% | UTCC  ≥17.0% | *p* value |
| --- | --- | --- | --- |
| Variables | (n = 1218) | (n = 735) |  |
| Age, y | 59.2 ± 15.7 | 63.3 ± 14.3 | <0.001 |
| Male, n (%) | 809 (66.4) | 388 (52.8) | <0.001 |
| Body mass index, kg/m^2^ | 23.9 ± 3.7 | 24.4 ±4.2 | 0.007 |
| Systolic blood pressure, mm Hg | 131.8 ± 18.1 | 133.2 ± 19.2 | 0.10 |
| Diastolic blood pressure, mm Hg | 78.8 ± 12.3 | 78.7 ± 12.7 | 0.85 |
| Heart rate, bpm | 64.2 ± 8.5 | 76.6 ± 12.6 | <0.001 |
| Total cholesterol, mmol/L | 4.96 ± 0.92 | 4.98 ± 1.03 | 0.75 |
| Triglycerides, mmol/L | 1.55 ± 1.23 | 1.68 ± 1.25 | 0.03 |
| HDL cholesterol, mmol/L | 1.55 ± 0.43 | 1.52 ± 0.44 | 0.26 |
| LDL cholesterol, mmol/L | 2.89 ± 0.81 | 2.85 ± 0.87 | 0.31 |
| Glucose, mmol/L | 6.11 ± 1.86 | 6.44 ± 2.04 | <0.001 |
| HbA1c, % | 5.8 ± 0.9 | 6.0 ± 0.8 | 0.003 |
| Smoking, n (%) | 656 (54.2) | 368 (50.3) | 0.10 |
| Comorbidities |  |  |  |
| Hypertension, n (%) | 984 (80.8) | 634 (86.3) | 0.002 |
| Dyslipidemia, n (%) | 823 (67.6) | 570 (77.6) | <0.001 |
| Diabetes mellitus, n (%) | 263 (21.6) | 245 (33.3) | <0.001 |
| Coronary artery disease, n (%) | 125 (10.3) | 115 (15.7) | <0.001 |
| Prior coronary intervention, n (%) | 97 (8.0) | 93 (12.7) | <0.001 |
| Cerebrovascular disease, n (%) | 70 (5.8) | 60 (8.2) | 0.04 |
| Hemodialysis, n (%) | 3 (0.3) | 10 (1.4) | 0.004 |
| Medications, n (%) |  |  |  |
| Antihypertensive drugs | 793 (65.1) | 536 (72.9) | <0.001 |
| Lipid-lowering drugs | 395 (32.4) | 306 (41.6) | <0.001 |
| Antidiabetic drugs | 160 (13.1) | 193 (26.3) | <0.001 |

%UTCC indicates upstroke time per cardiac cycle; bpm, beats per minute; HDL, high-density lipoprotein; LDL, low-density lipoprotein; HbA1c, hemoglobin A1c.

**Supplementary Table 7**. Association between coronary artery disease and upstroke time per cardiac cycle

|  | Odds ratio (95% Confidence interval);  *p* value | | |
| --- | --- | --- | --- |
| Variable | Unadjusted | Model 1^a^ | Model 2^b^ |
| UTCC <17.0% | 1 (reference) | 1 (reference) | 1 (reference) |
| UTCC ≥17.0% | 1.62 (1.24–2.13);  <0.001 | 1.58 (1.18–2.11);  0.002 | 1.46 (1.07–1.99);  0.02 |
| Every 1-SD (3.18%) increment of UTCC | 1.28 (1.13–1.45);  <0.001 | 1.22 (1.06–1.39);  0.005 | 1.14 (0.98–1.32);  0.08 |

^a^Model 1: adjusted for age and sex.

^b^Model 2: adjusted for age, sex, body mass index, hypertension, low-density lipoprotein cholesterol, diabetes mellitus, and smoking.

UTCC indicates upstroke time per cardiac cycle; SD, standard deviation.

**Supplementary Figure 1**


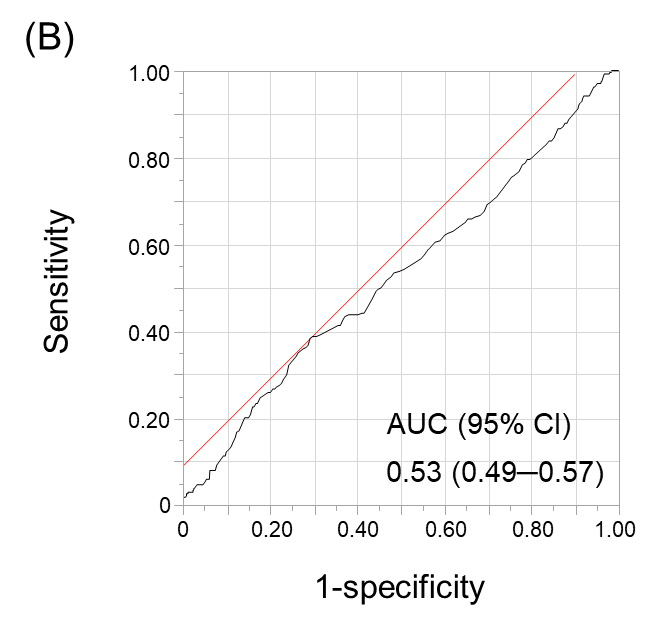

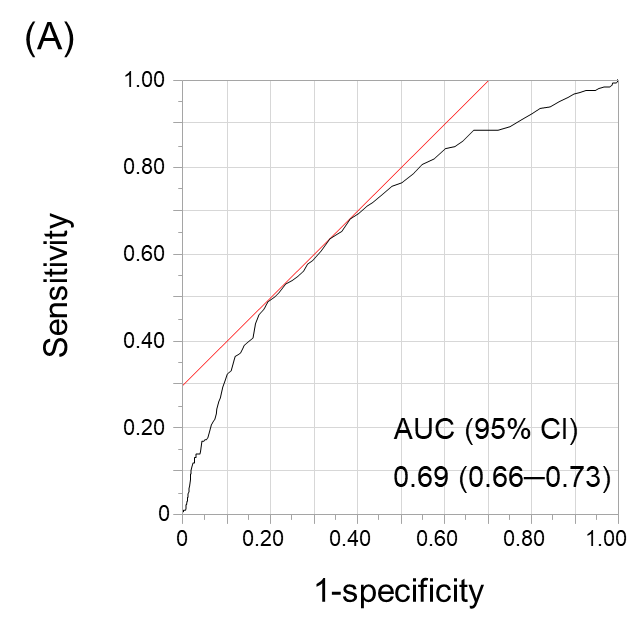


Receiver operating characteristic curves of upstroke time (A) and percentage of mean arterial pressure (B) to diagnose patients with clinical coronary artery disease among subjects with normal ankle-brachial index. AUC indicates area under the curve; CI, confidence interval.

**Supplementary Figure 2**

100

150

200

250

30

35

40

45

50

Upstroke time (ms)

%MAP (%)

Group 1

Group 2

Group 3

Group 4

Scatter plots based on upstroke time and percentage of mean arterial pressure (%MAP).
